# Arsenic in Drinking Water and Peripheral Nerve Conduction Velocity among Residents of a Chronically Arsenic-affected Area in Inner Mongolia

**DOI:** 10.2188/jea.16.207

**Published:** 2006-09-04

**Authors:** Yoshihisa Fujino, Xiaojuan Guo, Kiyoyumi Shirane, Jun Liu, Kegong Wu, Munetoshi Miyatake, Kimiko Tanabe, Tetsuya Kusuda, Takesumi Yoshimura, Japan Inner Mongolia Arsenic Pollution Study Group.

**Affiliations:** 1 Department of Preventive Medicine and Community Health, University of Occupational and Environmental Health, Japan; 2 Department of Preventive Medicine, School of Public Health, Wenzhou Medical College.; 3 Department of Clinical Epidemiology, Institute of Industrial Ecological Science, University of Occupational and Environmental Health, Japan; 4 Center for Endemic Disease Control and Research of Inner Mongolia.; 5 Department of Applied Chemistry, Faculty of Engineering, Miyazaki University.; 6 Materials Research Center, Miyazaki University.; 7 Department of Urban and Environmental Engineering, Graduate School, Kyushu University.; 8 Fukuoka Institute of Health and Environmental Sciences.; 9 See appendix for the investigators (name and affiliation) involved in the Japan Inner Mongolia Arsenic Pollution Study Group.

**Keywords:** Arsenic, Drinking, Electromyography, Neural Conduction, China

## Abstract

**Background:**

It remains unclear whether chronic ingestion of arsenic in drinking water affects the peripheral nervous system. We examined the effects of arsenic exposure on nerve conduction velocity using electromyography.

**Methods:**

A cross-sectional study was conducted of a population living in an arsenic-affected village in Hetao Plain, Inner Mongolia, China. A total of 134 (93.7%) of 143 inhabitants took part in the study, and 36 (76.6%) of 47 inhabitants in a low-arsenic exposed village were recruited as a control group. Of the participants, 109 inhabitants in the arsenic-affected village and 32 in the low-arsenic exposed village aged ≥18 years were used for the analyses. An expert physician performed skin examinations, and median nerve conduction velocity was examined by electromyography. Arsenic levels in tube-well water and urine were measured. A mean level of arsenic in tube-well water in the arsenic-affected village was 158.3*μ*g/L, while that in the low-arsenic exposed village was 5.3*μ*g/L.

**Results:**

No significant differences in the means of the motor nerve conduction velocity (MCV) and sensory nerve conduction velocity (SCV) were observed in relation to arsenic levels in tube wells, urine, and the duration of tube-well use. Further, no differences in mean MCV or SCV were found between the subjects with and without arsenic dermatosis, with mean SCV of 52.8 m/s (SD 6.3) in those without and 54.6 m/s (5.2) in subjects with arsenic dermatosis (p=0.206).

**Conclusion:**

These findings suggest that chronic arsenic poisoning from drinking water is unlikely to affect nerve conduction velocity, at least within the range of arsenic in drinking water examined in the present study.

Chronic arsenic exposure from drinking water is recognized as one of the most serious public health issues in many countries. Large numbers of people throughout the world in countries including Argentina, Bangladesh, Chile, India, China, and the USA have been exposed to arsenic by this route.^1^^,^^2^ The most common adverse effects are skin changes including hyperkeratosis, hyperpigmentation, and depigmentation.^1^^,^^2^ Over time, arsenic ingestion can cause cancers in various sites including the skin, bladder, liver, and lung.^1^^,^^3^^,^^4^ It is known that acute arsenic poisoning has a neurotoxic effect on both the central and peripheral nervous systems. Sensory nerves are particularly likely to be affected. Several studies that examined nerve conduction velocity using electromyography reported that conduction is slowed or prolonged in response to arsenic poisoning, while histopathological examination has revealed Wellerian degeneration and sensorimotor axonopathy.^5^

Additionally, the neurotoxic effect of chronic arsenic poisoning via contaminated drinking water has also been investigated in a number of epidemiologic studies, but several of these reported peripheral neuropathy as indicated by clinical symptoms only. To our knowledge, however, only a few studies have used electromyography to examine the neurotoxic effect of chronic arsenic exposure, and the results have been inconsistent.^6^^-^^9^ Specifically, Hindmarsh et al^9^ reported a positive association between abnormalities on electromyography and the level of arsenic in well water (range 60 to 1400*μ*g/L). In contrast, Kreiss et al^8^ reported no significant increase in abnormal nerve conduction velocity among subjects who drank arsenic-contaminated water (mean 347*μ*g/L; range 1 to 4780*μ*g/L), while Southwick et al^6^ reported no significant difference in nerve conduction velocity between controls and exposed subjects in two communities (average water arsenic concentration 180*μ*g/L and 270*μ*g/L). Moreover, no study has examined the neurotoxic effect of chronic arsenic poisoning by electromyography among residents of Inner Mongolia, one of the most seriously affected areas. The effect of chronic arsenic poisoning on the peripheral nervous system thus remains unclear.

Here, we examined the effect of chronic arsenic poisoning by electromyography in a population in Inner Mongolia with chronic ingestion of arsenic in drinking water.

## METHODS

### Study Population

The study was conducted in September 2002 among the populations of two villages supplied by tube-well water in Wuyuan Prefecture, Hetao Plain, Inner Mongolia, one with high and the other with low levels of arsenic. Both villages are isolated and supported by subsistence agriculture, with no industry or mining in the area. The villagers supplement their income by selling farm products. In the arsenic-affected village, drinking water was obtained from four open-shallow public wells (depth 3-5 m) which did not contain arsenic until the 1970s. Since 1980, however, most of the villagers have used private household tube wells (depth 15-30 m) installed for hygiene purposes. Drinking water is now obtained exclusively from tube wells in both villages. Unfortunately, however, ground water in tube wells was subsequently found to contain high levels of arsenic in the late 1980s.

A total of 134 (93.7%) of the 143 inhabitants in the arsenic-affected village and 36 (76.6%) of 47 in the low-arsenic exposed village participated in the study. All villagers were engaged in agriculture except for two in the arsenic-affected village and one in the low-arsenic exposed village. The total number of inhabitants in each village was calculated using records registered in the local government office in Wuyuan Prefecture. The analysis was limited to 109 subjects in the arsenic-affected village and 32 in the low-arsenic exposed village aged 18 years or older, on the basis of uncertainties regarding the susceptibility of children to arsenic. Children consume much more water than adults on a body-weight basis and are regarded as more susceptible to arsenic poisoning, while adults are generally exposed for longer than children.^10^ Written informed consent was obtained from the participants after local government personnel had explained the study purpose and invited participation. The project was approved by the ethics committee of the University of Occupational and Environmental Health, Japan.

### Interviews and Skin Examination

Subjects were invited to participate in a survey enquiring about socio-demographic conditions, residential history, duration of tube-well use, occupation, working conditions, and health-related lifestyle habits such as smoking and alcohol consumption. After the interview, the subjects were also invited to undergo a skin examination by an expert physician. Subjects who had obvious skin abnormalities, including hyperkeratosis of the palms and soles, or hyperpigmentation or depigmentation of the torso and limbs were diagnosed as having arsenic dermatosis.^11^^,^^12^ At the time of examination, the physician was blinded to the answers obtained during interview.

To avoid observation bias, all subjects in both villages were examined by the same physician and interviewer. The subjects in the arsenic-affected village were generally aware that the water was contaminated, but neither subjects nor investigators were informed about the arsenic level in their particular tube well until the study was completed.

### Examination of Nerve Conduction Velocity

Motor nerve conduction velocity (MCV) or sensory nerve conduction velocity (SCV) in the median nerve were examined using a Neuro Tester NRS1100 (Aster Electric Co., Ltd., Japan) in accordance with standard procedure.^13^^,^^14^ Room temperature was maintained at 22 to 25°C. The recording surface electrode was placed over the abductor pollicis brevis muscle, and the indifferent electrode was placed just distal to the metacarpophalangeal joint of the thumb. The median nerve was stimulated supramaximally at the wrist, 7 cm proximal to the recording electrode, and at the elbow by two electrodes placed longitudinally over the median nerve. The compound muscle action potential was recorded, and motor distal latencies to onset were measured. Median motor nerve conduction velocity was calculated by dividing the distance between the stimulation point at the wrist and at the elbow by the latency difference between the wrist and elbow stimulation. Median sensory nerve action potentials were determined by antidromic stimulation at the wrist, with the recording electrodes placed over the medial phalanx of the right index finger. Supramaximal responses were obtained, and sensory latencies to onset were measured on wrist stimulation. All sensory responses were averaged five times to obtain clear onset latencies. SCV was calculated by dividing the distance between the stimulating electrode at the wrist and the recording electrodes by the measured latency.

### Analysis of Arsenic in Urine and Tube-well Water

First morning urine samples collected from the study participants and water samples collected directly from tube wells were frozen immediately after collection and used for determination of arsenic concentrations.

Quantitation of arsenic in water and urine was performed as reported previously.^15^ Briefly, 0.20 mL portions of urine were transferred into 20 mL polypropylene test tubes and mixed with 0.80 mL 2 M sodium hydroxide, and then the mixture was heated at 95°C for 3 hours. These treated urine samples and untreated tube-well water samples were diluted to make a 2 mL sample. Arsenic levels were determined using an atomic absorption spectrophotometer incorporating an Arsenic Speciation Pretreatment System (Model No: AA-6800, ASA-2sp; Shimadzu Corporation, Kyoto, Japan). The standard reference materials used were Arsenic Standard Solution (Wako Pure Chemical Industries, Ltd).

### Statistical Analysis

Analysis of variance was performed to compare the means of MCV or SCV according to the level of arsenic in the tube wells (arsenic <50*μ*g/L, 50-99*μ*g/L, 100-150*μ*g/L, and >150*μ*g/L) and in urine (arsenic <170*μ*g/g creatinine, 170-439*μ*g/g creatinine, 440-570, and >570*μ*g/g creatinine). Analysis was also performed according to the duration of tube-well use (duration of tube-well use <6 years, 6-14.9 years, 15-18.9 years, and ≥19 years). Means of MCV or SCV were also compared for subjects with or without arsenic dermatosis. Further analyses of covariance were conducted for age, sex, duration of tube-well use, level of arsenic in the tube well, and level of arsenic in urine to compare the means of MCV or SCV. Analyses were performed using the subjects combining with tow villages. Calculations were per-formed using the SAS^®^.^16^

## RESULTS

A total of 46 of 49 tube wells in the arsenic-affected village were contaminated to a level above 100*μ*g/L, with a mean level in this location of 158.3*μ*g/L (SD=24.7) and a maximum level 197.3*μ*g/L. Levels in the second village approximately 10 kilometers away were particularly low, with 15 of 19 wells containing less than the WHO water quality standard of 10*μ*g/L(17) and the remaining 4 containing less than 20*μ*g/L. Mean level was 5.3*μ*g/L (SD=5.2) and maximum was 18.7*μ*g/L.

Overall, 63% of male subjects and 48% of female ones in the affected villages had arsenic dermatosis (Table 1). Dermatosis was more likely in those subjects who were still exposed to high levels of arsenic in water (Table 2). Regarding neuroconduction, in contrast, no significant difference between the groups was seen in mean MCV or SCV by arsenic level in the wells. Further, no difference was seen by levels in urine (Table 3), which incidentally showed a significant correlation to water levels (Pearson correlation coefficient = 0.78, p < 0.001). Moreover, no significant difference in mean MCV or SCV was seen by duration of tube-well use (Table 4).

**Table 1.** Characteristics of the subjects and neuroconduction velocity according to the resident areas.

	low-arsenic village		arsenic-affected village	
	
	male	female	male	female
	n=16	n=16	n=59	n=50
Mean age (SD)	43.1 (11.1)	41.0 (9.2)	43.4 (13.7)	42.8 (13.3)
Arsenic level in tube-well water(*μ*g/L)	6.6 (5.3)	4.8 (4.9)	149.7 (37.7)	150.8 (34.3)
Arsenic level in urine (*μ*g/g creatinine)	37.1 (17.1)	19.2 (8.7)	503.3 (165.6)	531.0 (221.8)
Current smoker (%)	94	0	66	12
Arsenic dermatosis (%)	0	0	63	48

Mean motor nerve conduction velocity (m/s) (SD)	55.6 (3.5)	55.5 (4.4)	55.8 (5.2)	57.0 (4.5)
Mean sensory nerve conduction velocity (m/s) (SD)	53.3 (5.9)	50.0 (6.0)	54.7 (5.2)	53.7 (6.1)

**Table 2.** Characteristics of the subjects and neuroconduction velocity according to the level of arsenic in tube-well.

	Arsenic level in tube-well water (*μ*g/L)				P*	P^†^

	<50	50-99	100-149	150-200		
	n=33	n=6	n=30	n=70		
Mean age (SD)	42.4 (10.7)	48.0 (23.2)	40.2 (13.0)	44.0 (12.6)		
Arsenic level in urine (*μ*g/g creatinine)	64.8 (119.8)	445.6 (241.4)	419.9 (193.0)	567.1 (175.2)		
Male sex (%)	52	50	53	54		
Current smoker (%)	53	33	33	43		
Arsenic dermatosis (%)	3	33	50	61		
Mean duration of tube-well use (year) (SD)	12.5 (7.6)	14.2 (7.5)	8.9 (6.2)	15.3 (5.8)		

Mean motor nerve conduction velocity (m/s) (SD)	55.7 (3.9)	56.0 (2.5)	55.6 (3.3)	56.7 (5.6)	0.690	0.602
Mean sensory nerve conduction velocity (m/s) (SD)	51.7 (6.0)	56.5 (2.7)	53.3 (3.2)	54.4 (6.5)	0.137	0.125

* : P values were derived from one-way analysis of variance.

† : P values were derived from analysis of covariance. The model included age and sex.

**Table 3.** Characteristics of the subjects and neuroconduction velocity according to the level of arsenic in urine.

	Arsenic level in urine (*μ*g/g creatinine)				P*	P^†^

	< 170	170 - 439	440 - 570	> 570		
	n=36	n=36	n=33	n=36		
Mean age (SD)	41.1 (9.9)	41.1 (13.4)	43.4 (10.7)	45.9 (15.8)		
Arsenic level in tube-well water (*μ*g/L)	19.6 (41.1)	141.5 (36.1)	146.8 (41.3)	165.0 (24.0)		
Male sex (%)	50.0	52.8	60.6	50.0		
Current smoker (%)	45.71	38.89	54.55	33.33		
Arsenic dermatosis (%)	2.8	47.2	63.6	61.1		
Mean duration of tube-well use (year) (SD)	11.0 (7.7)	12.2 (6.1)	13.6 (7.7)	15.4 (5.3)		

Mean motor nerve conduction velocity (m/s) (SD)	56.0 (4.0)	56.0 (4.2)	56.3 (6.1)	56.5 (4.3)	0.970	0.934
Mean sensory nerve conduction velocity (m/s) (SD)	52.3 (6.1)	53.4 (5.5)	54.0 (6.3)	54.9 (5.3)	0.392	0.934

* : P values were derived from one-way analysis of variance.

† : P values were derived from analysis of covariance. The model included age and sex.

**Table 4.** Characteristics of the subjects and neuroconduction velocity according to the duration of exposure.

	Duration of tube-well use (years)				P*	P^†^

	< 6	6 - 14.9	15 - 18.9	≥ 19		
	n=33	n=38	n=31	n=39		
Mean age (SD)	40.5 (13.5)	37.4 (9.8)	43.9 (11.5)	49.4 (12.9)		
Male sex (%)	45	55	55	56		
Current smoker (%)	41	39	55	38		
Arsenic dermatosis (%)	36	32	55	51		
Mean levels of arsenic in tube-well water (*μ*g/L) (SD)	107.7 (65.2)	100.8 (67.7)	145.5 (57.8)	124.8 (71.7)		
Arsenic level in urine (*μ*g/g creatinine)	312.9 (234.9)	341.3 (259.5)	532.7 (268.2)	444.7 (256.6)		

Mean motor nerve conduction velocity (m/s) (SD)	55.9 (3.8)	55.6 (3.1)	56.8 (6.2)	56.5 (5.4)	0.736	0.441
Mean sensory nerve conduction velocity (m/s) (SD)	53.5 (5.4)	53.2 (4.9)	54.4 (6.2)	53.4 (6.9)	0.857	0.672

* : P values were derived from one-way analysis of variance.

† : P values were derived from analysis of covariance. The model included age and sex.

Subject characteristics and their nerve conduction velocity according to arsenic dermatosis status are shown in Table 5. Those with arsenic dermatosis were slightly older and had been exposed for longer. There were no significant differences in mean MCV or SCV between the groups.

**Table 5.** Characteristics of the subjects and neuroconduction velocity according to the arsenic dermatosis.

	arsenic dermatosis		P*	P^†^

	without	with		
	n=80	n=61		
Mean age (SD)	40.5 (14.0)	46.1 (10.0)		
Male sex (%)	48	61		
Current smoker (%)	42	44		
Mean duration (year) of tube-well use (SD)	12.3 (6.9)	14.0 (6.8)		
Mean levels of arsenic in tube-well water (*μ*g/L) (SD)	91.0 (75.3)	154.8 (30.6)		
Arsenic level in urine (*μ*g/g creatinine)	310.7 (291.4)	529.4 (160.9)		

Mean motor nerve conduction velocity (m/s) (SD)	56.4 (4.3)	55.9 (5.1)	0.603	0.495
Mean sensory nerve conduction velocity (m/s) (SD)	52.8 (6.3)	54.6 (5.2)	0.093	0.206

* : P values were derived from t-test.

† : P values were derived from analysis of covariance. The model included age and sex.

Further, multivariate analyses of covariance show no associations between MSV or SCV and age, sex, duration of tube-well use, arsenic level in the tube wells, and arsenic level in urine (Table 6).

**Table 6.** Analyses of covariance* for motor nerve conduction velocity and sensory nerve conduction velocity.

	Coefficient	P
Motor nerve conduction velocity (m/s)		
Age	-0.051	0.262
Sex^†^	0.854	0.331
Duration of tube-well use (year)	0.066	0.335
Arsenic level in tube-well water (*μ*g/L)	0.002	0.848
Arsenic level in urine (*μ*g/g creatinine)	0.001	0.673

Sensory nerve conduction velocity (m/s)		
Age	-0.087	0.119
Sex^†^	-1.497	0.157
Duration of exposure (year)	0.052	0.528
Arsenic level in tube-well (*μ*g/L)	0.005	0.663
Arsenic level in urine (*μ*g/g creatinine)	0.003	0.387

* : The model included age, sex, duration of tube-well use, the levels of arsenic in tube-well and the levels of arsenic in urine.

† : Sex was coded as 0 for male, and as 1 for female.

## DISCUSSION

Our study of the neurotoxic effects of chronic exposure to arsenic in drinking water showed that MCV and SCV were both generally within the normal range in exposed subjects,^18^ and that mean MCV and SCV did not significantly differ by duration of tube-well use, status of arsenic dermatosis, or arsenic level in the tube wells or in urine.

Although peripheral neuropathy is a common sequela of acute arsenic poisoning, it remains unclear whether the chronic ingestion of arsenic in drinking water affects the peripheral nervous system. Several reports have found that ingestion of low levels in water is associated with peripheral neuropathy.^7^^,^^19^^,^^20^ Conversely, however, a review by Hotta et al^21^ reported that peripheral neuropathy was not a common or significant finding in studies of cohorts affected by chronic arsenic dermatitis caused by well water in Taiwan, Argentina, and Chile.

These studies used various methods to assess neurological dysfunction. Several of these studies used clinical symptoms only, and few studies have actually examined nerve conduction velocity, notwithstanding that nerve conduction velocity testing represents the only objective measurement of effects.

Among those using electromyogtaphy, Hindmarsh et al^9^ examined nerve abnormalities among 32 individuals in a Canadian community exposed to arsenic (range 60 to 1400*μ*g/L) in comparison to 12 control subjects, and found that higher levels of consumption correlated with an increased prevalence of abnormalities. Kreiss et al^8^ conducted a cross-sectional study of 147 residents of an arsenic-affected area (well arsenic levels ranging from 1 to 4781*μ*g/L) divided into three groups according to exposure level, but found no detectable differences in nerve conduction velocity. Southwick et al^6^ examined neurological data for the residents of Millard Country, Utah, USA, and showed no difference in prevalence of nerve abnormality between the exposed subjects and the controls. Thirteen of 83 exposed individuals and 8 of 67 controls had detectable signs of nerve abnormality. Mean nerve conduction velocity (NCV) was not significantly different in the exposed subjects compared to the controls, nor was annual arsenic dose associated with NCV by regression analysis.

Among other studies, Mukherjee et al^7^ recently reported electromyographic data for 55 subjects with arsenic dermatosis in West Bengal and Bangladesh exposed to drinking water levels of 100 to 700*μ*g/L. Neuropathy was diagnosed by ‘clinical neurological examination’. A total of 22 subjects experienced ‘sensory nerve dysfunction’, but nerve conduction velocity data were not provided. Problems with the previous studies should also be considered, including inadequate subject numbers,^9^ lack of external control groups,^7^^,^^8^ or overall lack of detailed data. Further, internal comparisons (comparison within the exposed village between high- and low-exposure subjects) might underestimate the effects of exposure, particularly when the dose-response relationship is assumed to be low.

Here, given previous reports that chronic arsenic poisoning caused peripheral neuropathy in a stocking and glove distribution, we examined NCV of the median nerve using electromyography.^7^^,^^8^^,^^22^ The results showed no effect of arsenic exposure on NCV in the present subjects. Of particular note, no difference was seen in mean SCV or MCV among the subjects with and without arsenic dermatosis, notwithtanding that skin abnormalities are a hallmark of chronic arsenic poisoning. Subjects with arsenic dermatosis might be more susceptible to this compound, possibly as a result of individual variations in arsenic metabolism, such as in methylating enzyme activity.^23^

These present results suggest that arsenic poisoning is unlikely to affect peripheral nerve conduction velocity, at least within the exposure levels and durations of ingestion examined in this study. Given that the range of levels in the tube-well water was relatively narrow, our discussion of a dose-response relationship is necessarily limited. A second limitation is that we did not examine other health conditions that may affect neurological status; one example might be carpal tunnel syndrome, given that the subjects were engaged in manual agricultural labor. Third, nerve conduction velocity measures are the only endpoint considered. Comparison with other clinical neurological examinations may increase the interpretability of these findings. Further, although the participation rate of 77% was acceptable, the sample size in the arsenic-free village was small, and it is uncertain whether this affected the results.

In conclusion, these results suggest that chronic arsenic poisoning from drinking water may not affect nerve conduction velocity, at least within the range of levels examined in the present study. However, the dose-response relationship remains to be ascertained, particularly at higher exposure levels.

## APPENDIX

Japan Inner Mongolia Arsenic Pollution (JIMAP) Study Group Investigators in the JIMAP study and their affiliations are as follows: Yoshihisa Fujino, Takesumi Yoshimura, Hiroshi Kasai, and Kiyoyumi Shirane, University of Occupational and Environmental Health, Japan; Tetsuya Kusuda, Kyushu University; Kimiko Tanabe and Munetoshi Miyatake; Miyazaki University; Liu Jun, Xia Yajuan, Wu Kegong, Li Yanhong, Guo Xiaojuan, Yi Liqi, Qin Yuexian, and You Lingui, Center for Endemic Disease Control and Research of Inner Mongolia, China; and Zhao Dongyue, Liu Jianguang, Qiao jiandong, Anti-epidemic Station of Wuyuan County, Inner Mongolia.
